# Mitochondrial DNA sequence analysis reveals multiple Pleistocene glacial refugia for the Yellow‐spotted mountain newt, *Neurergus derjugini *(Caudata: Salamandridae) in the mid‐Zagros range in Iran and Iraq

**DOI:** 10.1002/ece3.6098

**Published:** 2020-02-14

**Authors:** Maryam Malekoutian, Mozafar Sharifi, Somaye Vaissi

**Affiliations:** ^1^ Department of Biology Baghabrisham Razi University Kermanshah Iran

**Keywords:** demography, multiple glacial refugia, phylogeography patterns, pleistocene climatic fluctuations, population genetic structure

## Abstract

Phylogeography is often used to investigate the effects of glacial cycles on current genetic structure of various plant and animal species. This approach can also identify the number and location of glacial refugia as well as the recolonization routes from those refugia to the current locations. To identify the location of glacial refugia of the Yellow‐spotted mountain newt, *Neurergus derjugini,* we employed phylogeography patterns and genetic variability of this species by analyzing partial ND4 sequences (867 bp) of 67 specimens from 15 sampling localities from the whole species range in Iran and Iraq. Phylogenetic trees concordant with haplotype networks showed a clear genetic structure among populations as three groups corresponding to the populations in the north, center, and south. Evolutionary ages of clades north and south ranging from 0.15 to 0.17 Myr, while the oldest clade is the central clade, corresponding to 0.32 Myr. Bayesian skyline plots of population size change through time show a relatively slight increase until about 25 kyr (around the last glacial maximum) and a decline of population size about 2.5 kyr. The presence of geographically structured clades in north, center, and south sections of the species range signifies the disjunct populations that have emerged in three different refugium. This study illustrates the importance of the effect of previous glacial cycles in shaping the genetic structure of mountain species in the Zagros range. These areas are important in terms of long‐term species persistence and therefore valuable areas for conservation of biodiversity.

## Graphical abstract

To identify the location of glacial refugia of the Yellow‐spotted mountain newt, Neurergus derjugini, we employed phylogeography patterns and genetic variability of this species by analyzing partial ND4 sequences from the whole species range in Iran and Iraq. Phylogenetic trees concordant with haplotype networks showed a clear genetic structure among populations as three groups corresponding to the populations in north, center, and south. The presence of geographically structured clades by AMOVA analysis indicated the disjunct populations of N. derjugini may have been survived in three different glacial refugia in the Zagros mountains.

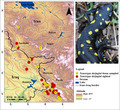

## 1 INTRODUCTION

Quaternary glacial–interglacial periods had a significant impact on the current pattern of species distribution and interpopulation gene flow (Fedorov & Stenseth, 2002; Ikeda et al., 2017). Generally, glacial plates at higher latitude (and elevation) made habitats inappropriate to inhabit, while climate at the same location at lower latitude (and elevation) was habitable (Ohlemüller, Huntley, Normand, & Svenning, 2012). Populations at higher latitude may cope through adaptation or phenotypic plasticity, or alternatively they may track suitable habitat (Hoffmann & Sgro, 2011). However, a more likely outcome is that such populations go extinct (Stewart, Lister, Barnes, & Dalén, 2009). At lower latitudes, populations can endure glacial periods relatively unaffected in so called glacial refugia (Bennett & Provan, 2008). Glacial refugium refers to a location where the mountain species can survived in the glacial periods, regardless of the geographical position or spatial extent of the location (Holderegger & Thiel‐Egenter, 2009). Biogeographic patterns are one approaches for identifying and describing refugia and suggesting that refugia existed in an area at some stage in the past (Keppel et al., 2012). Numerous studies are now revealing that within each of the main refugial areas, intraspecific lineages exhibited differential responses to climatic changes, often surviving in distinct, allopatric local refugia and exhibiting population fragmentation, allopatri differentiation, demographic expansion, and population admixture (Branco, Monnerot, Ferrand, & Templeton, 2002; García‐París, Alcobendas, Buckley, & Wake, 2003; Mattoccia, Marta, Romano, & Sbordoni, 2011). On a broader scale, in the case of widespread species a detailed knowledge of phylogeographic patterns within refugial areas is crucial for deriving robust inferences about the evolutionary history of species at a continental scale (Gómez & Lunt, 2007). On a finer scale, this approach depicts the existence of multiple refugial areas and the succession of events of population fragmentation, allopatric differentiation, demographic expansion, and population admixture (Sequeira, Alexandrino, Rocha, Arntzen, & Ferrand, 2005).

In higher latitudes in North American and Northern Europe, the influence of Quaternary climatic fluctuations on plant and animal species is well documented (Hewitt, 2011); however, such influence of climate change on range dynamics of species in other parts of the world is much less reported (Jandzik et al., 2018). Various geomorphic studies in Iran, including lake sediments, deserts, glacial moraines and periglacial features, salt domes, alluvial sediments, pediments and alluvial fans, fluvial and marine terraces, and loess‐soil sequences have shown that Iran was affected by Pleistocene and Holocene climate fluctuations (Kehl, 2009). The available paleoecological and palynological records in Iran indicate that the last glacial maximum (LGM) was characterized by a cooler and more arid climate compared to the Holocene (Djamali et al., 2012; Kehl, 2009). At the present time, deep valleys of several high altitude mountain areas in the Iranian Plateau are covered by glaciers. In the LGM, the expansion of such climatic conditions encouraged the spread of steppe habitats in lower altitudes, while high altitudes in the mountain areas were covered by glaciers (Ghahroudi Tali, Naeimi, & Gharnaie, 2016). In Zagros range, there are reports of isolated populations of various temperate evergreen plant species such as *Olea europaea* and *Myrtus communis* (Sharifi, Najafi, Yossefshahi, & Hemmati, 2001) and pockets of plant communities such as mires and patterned mires (Wasylikowa & Walanus, 2004), floating meadow and highland peatlands (Sharifi, Rezaii, Hosseini, & Raji, 2004). These relict individuals and plant communities are assumed to persist in enclaves of environmental conditions within an inhospitable regional climate and left behind during range shifts caused by Pleistocene climatic fluctuations (Hampe & Jump, 2011; Wright, McAndrews, & Van Zeist, 1967). According to Djamali et al. (2008), the vegetation composition during the LGM in the Lake Urmia, in northwestern Iran, demonstrates lower winter temperatures than today, and higher July temperatures about 11–12°C (Djamali et al., 2008).

The impact of climate fluctuations on several Iranian animal species has been investigated. These studies have shown that impact of climate fluctuation is more complicated and may not follow a prevailing scenario of glacial retraction and postglacial expansion as has been reported from the more northerly located regions of the western Palaearctic (Chiocchio, Bisconti, Zampiglia, Nascetti, & Canestrelli, 2017). Indeed, Southern Europe, Turkey, the southern parts of the Caucasus and Alborz act as multiple glacial refugia due to topographical heterogeneity and low latitude (Ahmadzadeh, Flecks, et al., 2013a; Asadi et al., 2019; Rossiter, Benda, Dietz, Zhang, & Jones, 2007). In Iran, some species experienced range contractions to glacial refugia located, for example, in the Zagros, Alborz, and Kope Dagh Mountains, which were followed by postglacial expansion for Brandt's Persian lizard, *Iranolacerta brandtii* (Ahmadzadeh, Carretero, et al., 2013b), oriental green lizards of the *Lacerta trilineata* (Ahmadzadeh, Flecks, et al., 2013a), two sympatric moth species, *Gnopharmia colchidari* and *G. kasrunensis* (Rajaei Sh et al., 2013), Persian jird, *Meriones persicus* (Dianat, Darvish, Cornette, Aliabadian, & Nicolas, 2017), greater horseshoe bat, *Rhinolophus ferrumequinum* (Shahabi, Akmali, & Sharifi, 2017), Asian pit viper, *Gloydius halys caucasicus* (Asadi et al., 2019), and Eastern rock nuthatch, *Sitta tephronota*, (Yousefi, Shabani, & Azarnivand, 2019). Some species did not change range such as Greek tortoise, *Testudo graeca* (Javanbakht et al., 2017), and Caspian turtles, *Mauremys caspica*, *M. rivulata* (Vamberger et al., 2017). There are even species that showed more extensive distribution during the glacial and retraction of ranges in interglacial as was evidenced for cold‐adapted species such as Yellow‐spotted mountain newt, *Neurergus derjugini* (Afroosheh et al., 2019), and Blanford's Semaphore gecko, *Pristurus rupestris* (Saberi‐Pirooz et al., 2019).

*Neurergus derjugini* is a critically endangered species occurring in 42 highland streams of the Zagros Mountain range in western Iran and eastern Iraq (Afroosheh, Akmali, Esmaeili‐Rineh, & Sharifi, 2016). This area is characterized by high physiographic complexity, with several large mountain ranges that in some area are large enough to facilitate precipitation and local open woodlands. In areas where mountains are enough large to prevent moist air parcels, similar oak forests develop in western–eastern corridors from northern Mesopotamian Plain to the western edge of the Iranian plateau. Within highland open woodland, 81% of the reported habitats for *N. derjugini* are located in Iran, 19% in Iraq (Afroosheh et al., 2016). The extent of occurrence of *N. derjugini* as indicated by a minimum convex polygon is 6,366 km^2^ for the 42 known localities (Afroosheh et al., 2016). This polygon is positioned along the western edge of the Zagros range with elevations ranging from 630 to 2057 masl. This area is covered by an oak open woodland. The breeding habitat of *N. derjugini* in the Zagros range has been degraded recently by water pollution, water extraction, and severe droughts, which have led to the extirpation of some populations (Afroosheh et al., 2016).

The present study analyzed spatial variation of mtDNA sequences, aiming to investigate the genetic structure within species, as well as attempting to trace the recent demographic and phylogeographical history of the *N. derjugini* in the Zagros range. We tested the multiple glacial refugia hypothesis for *N. derjugini* using molecular data in the whole specie range in mid‐Zagros range at the border of Iran and Iraq. We therefore aim to: (a) examine the extent of interspecific genetic variation and produce a phylogeography pattern of genetic diversity in various population of *N. derjugini,* (b) estimate time divergences among the different lineages, (c) determine if all populations derived from one glacial refugium or multiple refugia, (d) examine the similarity of the emerged pattern with the current subspecific taxonomy. Finally, we (e) match phylogeographic results with available Quaternary range dynamic outputs.

## 2 METHODS

### 2.1 Population sampling and sequencing

**Figure 1:**
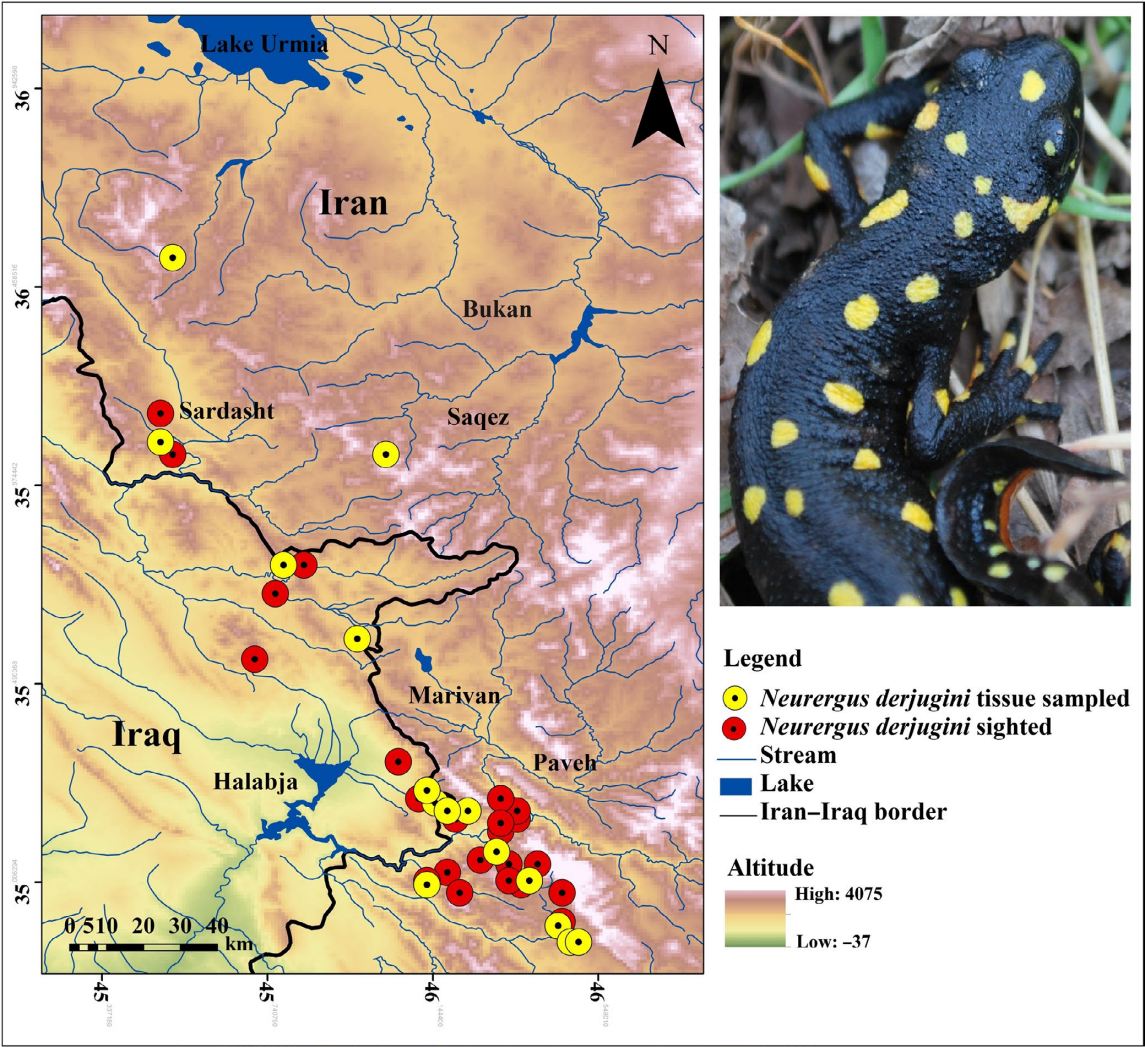
Geographical distribution of 42 populations of the Yellow‐spotted mountain newt, *Neurergus derjugini*, in Iran and Iraq

We were sampled from 67 individuals of *Neurergus derjugini* from 15 localities in Iran and Iraq during 2012–2014 (Table 1, Figure 1). Tissue samples were collected from each individual by removing a small section of tail or toe using sterile equipment. Tissue samples were stored in 95% ethanol at −20°C until DNA was extracted. Genomic DNA was extracted using tissue kit GenNetBio^TM^. We sequenced an 867 bp fragment of a mtDNA generation consisting of 726 bp fragment of NADH subunit 4, the whole tRNA‐His and tRNA‐Ser and 21 bp from the “5′” end of tRNA‐Leu using the primers ND4‐Leu: ND4, CAC CTA TGA CTA CCA AAA GCT CAT GTA GAA GC and Leu, CAT TAC TTT TAC TTG GAT TTG CAC CA (Arevalo, Davis, & Sites, 1994). Amplification of mtDNA was conducted using denaturation at 94°C for 2 min, 58°C for 45 s, 72°C for 2 min followed by 35 cycles at 94°C for 30 s, 58°C for 45 s, 72°C for 60 s for annealing and extension at 72°C for 3 min. Sequencing was performed by Microsynth Switzerland Laboratories.

**Table 1:** List of sampling locations used in this study and haplotypes with genetic diversities and frequencies

No.	Name of localities	Latitude (*N*)	Longitude (E)	Elevation (m)	Haplotypes and their frequencies	SS	H	Pi	Hd
1	Kavat	34° 52′ *N*	46° 30′ E	1601	Hap4(4)	4	1	0.00000	0.000
2	Ghorighale	34° 52′ *N*	46° 29′ E	1,600	Hap4(5)	5	1	0.00000	0.000
3	Gholani	34° 54′ *N*	46° 27′ E	1575	Hap4(3	3	1	0.00000	0.000
4	Dourisan	35° 01′ *N*	46° 23′ E	1,600	Hap4(4)	4	1	0.00000	0.000
5	Darrenajjar	35° 05′ *N*	46° 18′ E	1,472	Hap4(2), Hap5(1)	3	1	0.00076	0.666
6	Lashkargah	35° 00′ *N*	46° 08′ E	1,415	Hap4(5), Hap6(1)	6	2	0.00038	0.333
7	Nowdeshe	35° 11′ *N*	46° 14′ E	1,760	Hap7(2), Hap1(1)	3	2	0.00231	0.667
8	Hanigarmale	35° 14′ *N*	46° 08′ E	1,383	Hap2(4)	4	1	0.00000	0.000
9	Tawale	35° 11′ *N*	46° 11′ E	1,400	Hap1(2), Hap2(2)	4	2	0.00077	0.667
10	Balkha	35° 12′ *N*	46° 09′ E	1,482	Hap1(3), Hap2(1)	4	2	0.00058	0.500
11	Penjwin	35° 36′ *N*	45° 58′ E	1,421	Hap8(5)	5	1	0.00000	0.000
12	Siyagwez	35° 47′ *N*	45° 47′ E	1689	Hap8(6), Hap11(1)	7	2	0.00033	0.286
13	Shalmash	36° 05′ *N*	45° 29′ E	1622	Hap10(5)	5	1	0.00000	0.000
14	Saqez	36° 03′ *N*	46° 02′ E	2,168	Hap9(5)	5	1	0.00000	0.000
15	Benjun	36° 32′ *N*	45° 31′ E	2,152	Hap3(5)	5	1	0.00000	0.000
Total						67	11	0.00324	0.8304

Note

Abbreviation: SS, sample sizes; H, haplotypes and Pi, nucleotide diversity and Hd, haplotype diversity.

DNA sequences were aligned using the programs Clustal W in the BioEdit v.7.0.5.3 (Hall, 1999) and Muscle in MEGA 6 (Tamura, Stecher, Peterson, Filipski, & Kumar, 2013). As outgroups, we used *Neurergus kaiseri, N. strauchii, Triturus karelinii*, and *Ommatotriton vittatus* (GenBank accession numbers for outgroups: EU880320, EU880321, HQ697277, EU880338, respectively). Values for the numbers of haplotypes, polymorphic sites, parsimony informative site, haplotype diversity (Hd), nucleotide diversity (Pi) and number of transitions and transversions were calculated using the software Dnasp v 5.10.01 (Rozas, Librado, Sánchez‐Del Barrio, Messeguer, & Rozas, 2010) and Arlequin v 3.0 (Excoffier, Laval, & Schneider, 2005). To investigate pairwise sequences, divergence between haplotypes was computed using uncorrected Kimura 2‐parameter (K2P) model (Kimura, 1980) with 1,000 bootstrap replicates using the MEGA ver. 6 software package (Tamura et al., 2013). MtDNA haplotypes used in this study were deposited in the NCBI Nucleotide Database under accession numbers MN995069 to MN995079.

### 2.2 Phylogenetic analyses

Bayesian inference (BI) phylogeny was produced from the ND4 gene haplotypes by means of the program MrBayes, v 3.2.2 program (Ronquist et al., 2012) with 10,000,000 generations and Maximum likelihood (ML) by PhyML, v 3.0 program (Guindon et al., 2010) with 1,500 bootstrap replicates. Also we combined the ND4, ND2, and D‐loop sequences to reconstruct a maximum posterior probability tree using the program MrBayes 3.2.2 (Ronquist et al., 2012). ND2 and D‐loop sequences have been downloaded from GenBank as follows: accession numbers MK035716 – MK035726 for ND2 and MK098471 – MK098476 for D‐loop. The appropriate model for BI and ML analysis was selected with jModelTest v 0.1.1 program (Posada, 2008) with Akaike information criterion (AIC). The best fit model identified by AIC was TrN. The program FigTree v1.4.0 program (Rambaut, 2016) was used to visualize the phylogenetic tree. A nested clade phylogeographic analysis (NCPA) was constructed for ND4 using the TCS v 1.21 program (Clement, Posada, & Crandall, 2000).

### 2.3 Population analysis

Molecular variance was assessed using separate analyses of molecular variance (AMOVA) with 10,000 permutations in Arlequin version 3.0 (Excoffier et al., 2005). The analysis was performed considering populations from three geographical regions (northern, southern, and central haplogroups) to assess the level of genetic differentiation within populations. Then, all populations are considered as one group to assess the degree of differentiation among regions. Pairwise FST between populations was generated using Arlequin 3.0 (Excoffier et al., 2005).

### 2.4 Environmental data

We chose eight bioclimatic variables environmental characters of so evaluating by Sharifi, Karami, Akmali, Afroosheh, and Vaissi (2017). Bioclimatic variables included isothermality (BIO2/BIO7) (×100), temperature seasonality (BIO4, standard deviation ×100), temperature annual range (BIO5–BIO6), mean temperature of driest quarter (BIO9), mean temperature of wettest quarter (BIO8), precipitation of warmest quarter (BIO18), precipitation of coldest quarter (BIO19), and elevation. We used ArcMap 10.3 to process variables. The matrix of environmental distances was computed by SPSS version 16.0. We computed geographical distances using DIVA‐GIS v 7.5.0 (Hijmans, Guarino, & Mathur, 2004). We performed a Mantel test to evaluate correlations between genetic, geographical, and environmental distances using Arlequin 3.0 (Excoffier et al., 2005). This analysis runs with 10,000 random permutations. In addition, a three‐way Mantel test was applied between the matrix of pairwise genetic differentiation and the matrix of environmental distances and geographical distances among populations. We also performed principal components analysis (PCA) to investigate ecological differentiation within *N. derjugini* distribution range.

### 2.5 Demographic analysis

Mismatch distribution analysis was performed using DnaSP v 5.10.01 software (Rozas et al., 2010) to estimate population expansion as the distributions of the pairwise nucleotide differences. Haplotype and nucleotide diversity indices, Raggedness index, sum of squared deviations (SSD), and their variances as well as neutrality tests (Tajima's D, Fu's FS) were calculated using DnaSP v 5.10.01 software. The Bayesian skyline plots (BSP) produced from the ND4 gene haplotypes were obtained with a linear model using BEAST v 2.4.5 program (Bouckaert et al., 2014). Markov chain Monte Carlo technique (MCMC) run for 100 million generations sampled every 1,000 steps. Analysis performed with the uncorrelated lognormal relaxed clock and the Bayesian skyline as a coalescent model with the evolutionary rate for salamanders 0.64% per million years (Myr) per lineage (Malyarchuk, Derenko, & Denisova, 2013; Weisrock, Macey, Ugurtas, Larson, & Papenfuss, 2001). To estimate effective population size through time, Tracer v 1.6 (Rambaut, Suchard, Xie, & Drummond, 2016) was used.

### 2.6 Divergence time estimate

We estimate times of divergence between lineages of *N. derjugini* using BEAST v 2.4.5 (Bouckaert et al., 2014). We ran for 30,000,000 generations sampled every 1,000 steps, with the first 3,000,000 generations regarded as burn‐in 95% highest posterior density (95% HPD) were considered significant support. The maximum clade credibility tree summarized by Tree Annotator v1.8.4 program (Drummond & Rambaut, 2007). The calibration point was derived from molecular phylogenetic studies on the Salamandridae family estimate the divergence times, 0.64% per million years (Myr) per lineage, as proposed by Weisrock et al. (2001) and Malyarchuk et al. (2013). Sequences for outgroups have been downloaded as follows: *Triturus cristatus*: NC_015790; *T. dobrogicus*: HQ697274; *T. carnifex*: HQ697272; *T. marmoratus*; HQ697279; *T. pygmaeus*: HQ697280; *T. macedonicus*: HQ697278, in addition to the previous outgroups.

## 3 RESULTS

### 3.1 Genetic variation

We identified 11 haplotypes in a sample of 67 individuals of *N. derjugini* from 15 localities based on 863 base pairs of the mitochondrial gene, ND4. Haplotype diversity values range from 0 to 0.667. An indel occurred at the haplotype 5. Haplotype 4 was most widespread and abundant, shared among 6 of the 15 populations (Table 2). Eleven polymorphic sites were recorded including 11 transitions and 9 parsimony informative sites and one Indel site. Mean nucleotide composition were A: 31.79%, T: 30.24%, C: 23.44%, and G: 14.52%. Nucleotide diversity values varied from 0 to 0.00231. Pairwise uncorrected Kimura 2‐parameter of genetic distances among 15 populations of *N. derjugini* ranged from 0% to 0.04% (Table 3).

**Table 2:** Variable nucleotide positions and molecular diversity within the sequences (867 bp) of the ND4 for 11 haplotypes of 67 *Neurergus derjugini* sequences in different regions

Hap	Polymorphic site												Locality															
	328	337	376	398	493	576	580	583	634	646	695	779	Ka	Ghor	Ghol	Do	Da	La	No	Ha	Ba	Ta	Pe	Si	Sh	Sa	Be	Total
1	G	G	C	G	G	G	A	C	A	G	‐	C	0	0	0	0	0	0	1	0	3	2	0	0	0	0	0	6
2	.	.	.	.	.	.	.	.	G	.	‐	.	0	0	0	0	0	0	0	4	1	2	0	0	0	0	0	7
3	.	.	.	.	.	.	G	G	.	.	‐	.	0	0	0	0	0	0	0	0	0	0	0	0	0	0	5	5
4	.	.	.	.	A	A	.	T	.	.	‐	.	4	5	3	4	2	5	0	0	0	0	0	0	0	0	0	23
5	.	.	.	.	A	A	.	T	.	.	A	.	0	0	0	0	1	0	0	0	0	0	0	0	0	0	0	1
6	.	A	.	.	A	A	.	T	.	.	‐	.	0	0	0	0	0	1	0	0	0	0	0	0	0	0	0	1
7	A	.	.	.	.	A	.	T	.	.	‐	.	0	0	0	0	0	0	2	0	0	0	0	0	0	0	0	2
8	.	.	T	.	.	.	.	.	.	.	‐	.	0	0	0	0	0	0	0	0	0	0	5	6	0	0	0	11
9	.	.	.	.	.	.	G	.	.	A	‐	T	0	0	0	0	0	0	0	0	0	0	0	0	0	5	0	5
10	.	.	.	.	.	.	G	.	.	A	‐	.	0	0	0	0	0	0	0	0	0	0	0	0	5	0	0	5
11	.	.	T	A	.	.	.	.	.	.	‐	.	0	0	0	0	0	0	0	0	0	0	0	1	0	0	0	1
Sample size													4	5	3	4	3	6	3	4	4	4	5	7	5	5	5	67
Number of polymorphic sites													0	0	0	0	0	1	3	0	1	1	0	1	0	0	0	11
Number of transitions													0	0	0	0	0	1	3	0	1	1	0	1	0	0	0	11
Number of transversions													0	0	0	0	0	0	0	0	0	0	0	0	0	0	0	0
Number of parsimony informative sites													0	0	0	0	0	0	0	0	0	1	0	0	0	0	0	9
Average number of nucleotide difference													0	0	0	0	0	0.33	2	0	0.5	0.66	0	0.28	0	0	0	2.81

Abbreviations: Ka, Kavat; Ghor, Ghorighale; Ghol, Gholani; Do, Dourisan; Da, Darrenajjar; La, Lashkargah; No, Nowdeshe; Ha, Hani garmale; Ta, Tawella; Ba, Balkha; Pe, Penjwin; Si, Siya gwez; Sh, Shalmash; Sa, Saqez; Be, Benjun.

**Table 3:** K2P genetic distances values among 15 populations of *Neurergus derjugini.* Numbers are representative of localities as indicated in Table 1

**No**	**1**	**2**	**3**	**4**	**5**	**6**	**7**	**8**	**9**	**10**	**11**	**12**	**13**	**14**	**15**
1															
2	0.000														
3	0.000	0.000													
4	0.000	0.000	0.000												
5	0.000	0.000	0.000	0.000											
6	0.000	0.000	0.000	0.000	0.000										
7	0.000	0.000	0.000	0.000	0.000	0.000									
8	0.000	0.000	0.000	0.000	0.000	0.000	0.000								
9	0.000	0.000	0.000	0.000	0.000	0.000	0.000	0.000							
10	0.000	0.000	0.000	0.000	0.000	0.000	0.000	0.000	0.000						
11	0.000	0.000	0.000	0.000	0.000	0.000	0.000	0.000	0.000	0.000					
12	0.000	0.000	0.000	0.000	0.000	0.000	0.000	0.000	0.000	0.000	0.000				
13	0.000	0.000	0.000	0.000	0.000	0.000	0.000	0.000	0.000	0.000	0.000	0.000			
14	0.003	0.003	0.003	0.003	0.003	0.003	0.003	0.003	0.003	0.003	0.003	0.004	0.003		
15	0.000	0.000	0.000	0.000	0.000	0.000	0.000	0.000	0.000	0.000	0.000	0.000	0.000	0.003	0.000

### 3.2 Phylogenetic analyses

**Figure 2:**
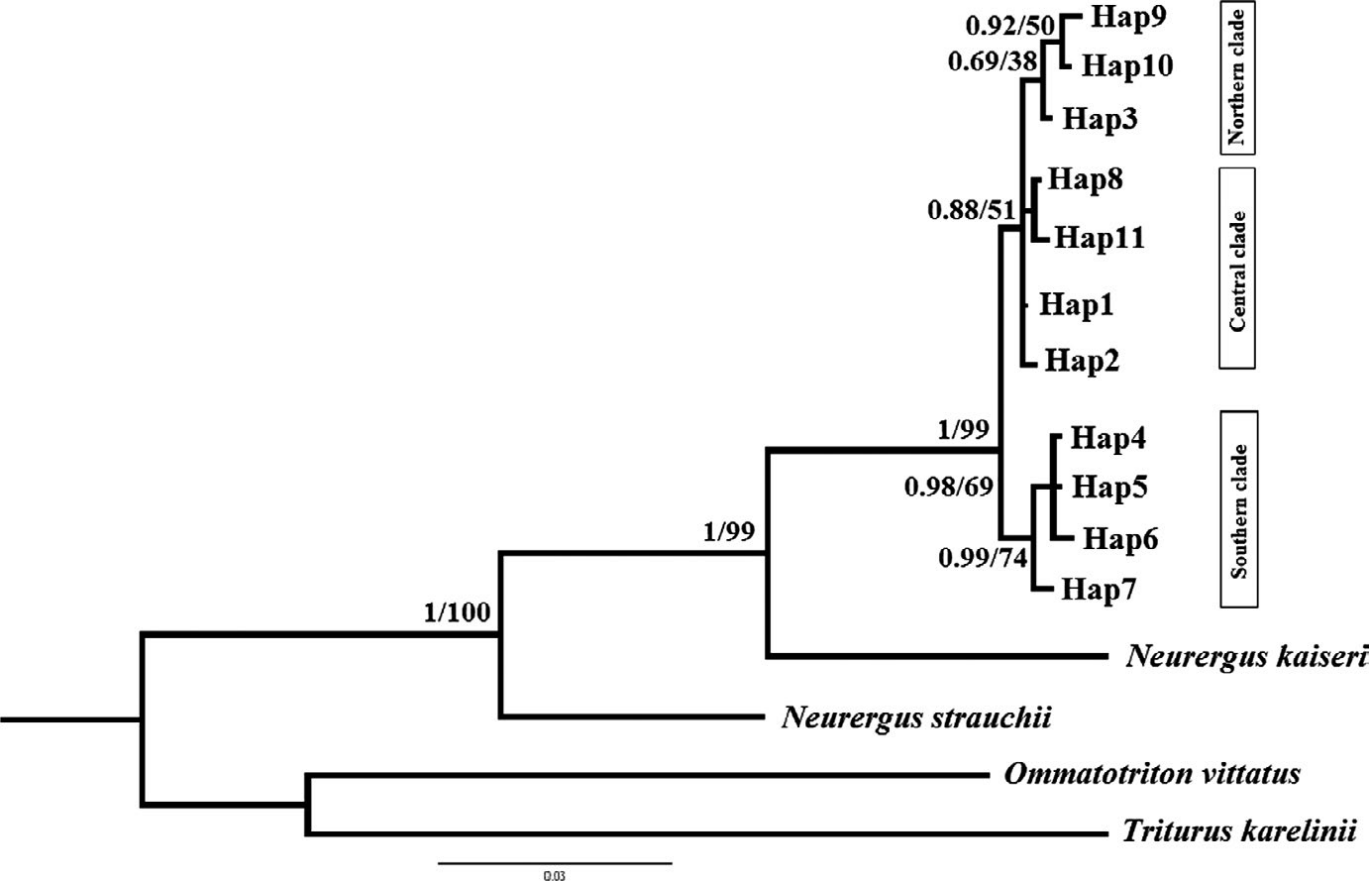
Phylogenetic trees of haplotypes implemented in PhyML and MrBayes based on partial ND4 gene sequence of 67 individuals for *Neurergus derjugini*. Bayesian posterior probability values are the left of the slash, and maximum likelihood bootstrap values are the right

**Figure 3:**
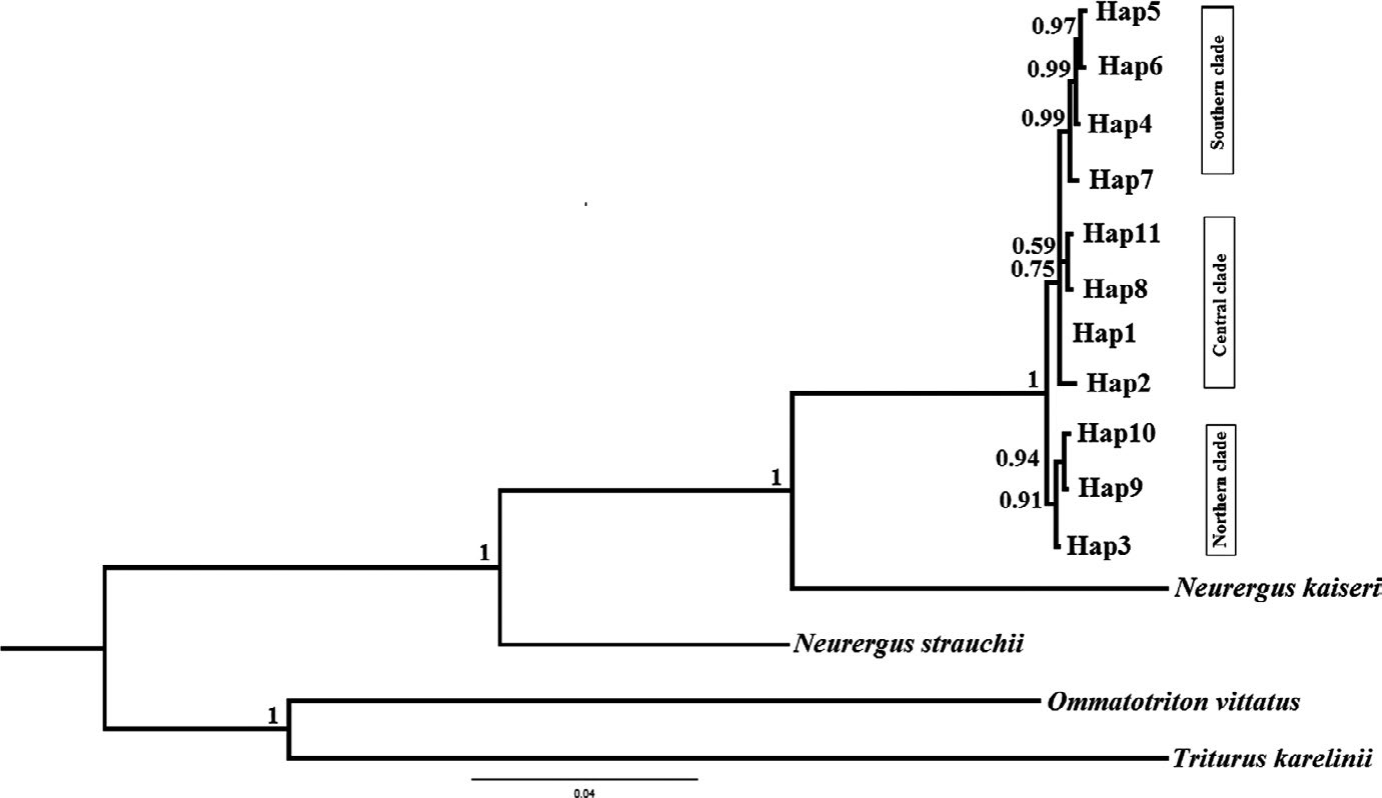
Phylogenetic tree of haplotypes implemented in MrBayes based on ND4, ND2, and D‐loop sequences for *Neurergus derjugini*. Numbers above branches indicate Bayesian posterior probability values

Phylogenetic relationships are represented by Bayesian and ML trees for 11 haplotypes of *N. derjugini* (Figure 2). Two tree topologies were similar. Monophyly of the *N. derjugini* haplogroups is well supported relative to the outgroup taxa (likelihood bootstrap 99%, posterior probability 1.00). In the phylogenetic trees recorded three groups corresponding to the populations north, center, and south (the northern: Shalmash, Saqez, Benjun; the central: Hani garmale, Tawella, Balkha, Penjwin, Siya gwez; and the southern: Kavat, Ghorighale, Gholani, Dourisan, Darrenajjar, Lashkargah, Nowdeshe), (Figure 2). *Neurergus derjugini* is well supported as sister taxon to *N. kaiseri* (likelihood bootstrap 99%, posterior probability 1.0). Within *N. derjugini*, there is little support for relationships northern populations sampled, with bootstrap 38%. The southern populations of *N. derjugini* from a well‐supported clade, with bootstrap 74%. To evaluate the phylogenetic analyses, 867 base pairs of ND4 sequences gene were combined with 718 base pairs of D‐loop and 1036 base pairs of ND2 sequeces gene. Based on the combined sequences, Bayesian inference trees showed that *N. derjugini* haplotypes form a monophyletic group with a three populations in north, center, and south (Figure 3).

**Figure 4:**
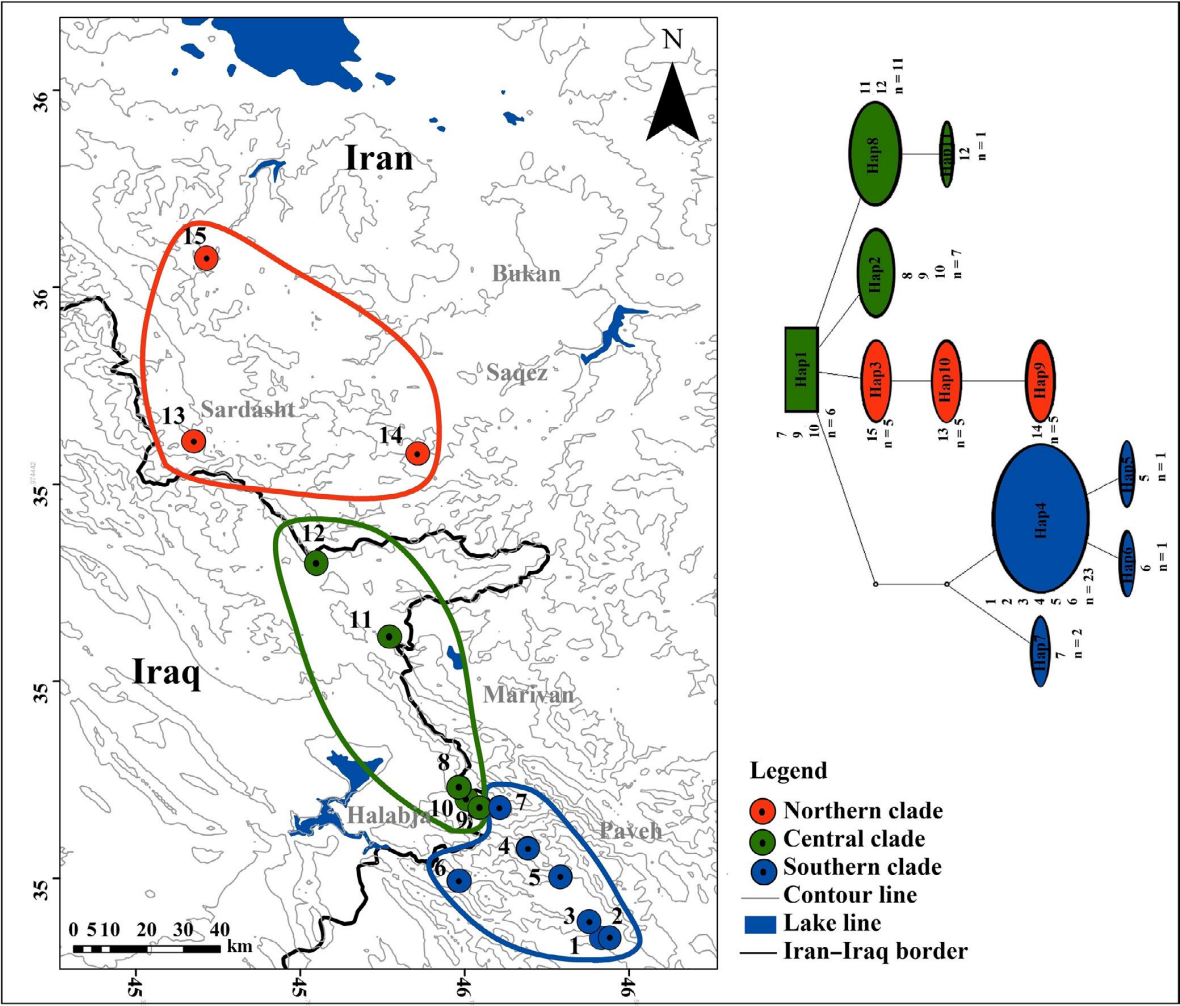
The map illustrates the sampling localities of *Neurergus derjugini* in Iran and Iraq, Haplotype network showing the phylogenetic relationships among the 11 haplotypes, Different haplotypes in the haplotype network have different colors, sizes of cycles are representative of the haplotype frequencies, and open dots represent missing intermediate haplotypes

The statistical parsimony haplotype network (Figure 4) suggested three haplotype subnetworks, which were in agreement with the topology described in the phylogenetic trees. The haplotype network pattern suggestive of little or no gene flow between regions because northern, central, and southern haplogroups were not intermingled.

### 3.3 Population analysis

The AMOVA analysis showed most genetic variation was significantly explained by differences among regions (74.87% genetic variation, *F*_CT_ = 0.74, *p* < .01), and genetic difference was detected among populations within regions (19.07 genetic variation, *F*_SC_ = 0.75, *p* < .001; Table 4). The genetic differences were significant among populations without the grouping. Most of the diversity was observed among populations (92.16%) and among regions (74.78%), while low percentage of variance was detected within populations (6.06%–7.84%; Table 4). The comparisons of pairs of population samples confirmed the high level of heterogeneity. Thirteen pairwise comparisons out of 105 (12.38%) were not significant (*p* < .05) indicating populations sharing the same haplotypes or closely related haplotypes. F_ST_ values between populations range from −0.166 to 1 (Table 5).

**Table 4:** Analysis of molecular variance (AMOVA) using partial ND4 gene

Structure	Source of variation	*df*	Variation (%)	F_SC_	F_ST_	F_CT_
Three regions	Among regions	2	74.87	0.75**	0.93**	0.74**
	Among populations within regions	12	19.07			
	Within populations	52	6.06			
The studied samples	Among populations		92.16			
	Within populations		7.84		0.92**	

Note

Significant values are shown for *p* < .05 “*” and *p* < .01 “**”.

**Table 5:** F_ST_ values between populations for ND4. Numbers are representative of localities as indicated in Table 1

**No**	1	2	3	4	5	6	7	8	9	10	11	12	13	14	15
1	0														
2	0	0													
3	0	0	0												
4	0	0	0	0											
5	0.111	0.189	0	0.111	0										
6	−0.081	−0.034	−0.153	−0.081	0.068	0									
7	0.641	0.690	0.571	0.641	0.5	0.648	0								
8	1	1	1	1	0.937	0.950	0.724	0							
9	0.904	0.917	0.886	0.904	0.826	0.874	0.506	0.333	0						
10	0.923	0.933	0.908	0.923	0.841	0.883	0.494	0.666	−0.166	0					
11	1	1	1	1	0.948	0.955	0.763	1	0.805	0.825	0				
12	0.954	0.958	0.948	0.954	0.913	0.928	0.753	0.912	0.742	0.739	−0.055	0			
13	1	1	1	1	0.957	0.964	0.825	1	0.884	0.903	1	0.945	0		
14	1	1	1	1	0.964	0.970	0.861	1	0.917	0.933	1	0.958	1	0	
15	1	1	1	1	0.948	0.955	0.763	1	0.805	0.825	1	0.920	1	1	0

### 3.4 Demographic analysis

**Figure 5:**
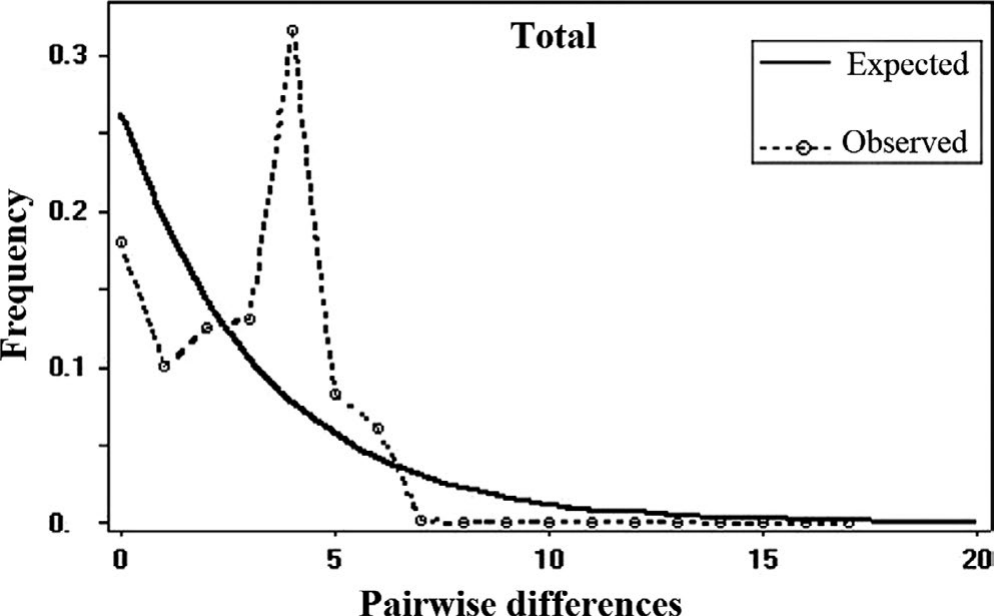
Mismatch distributions of the pairwise total population of *Neurergus derjugini*. The dashed lines represent the observed frequency of pairwise differences among sequences, and the lines show the expected distribution

**Figure 6:**
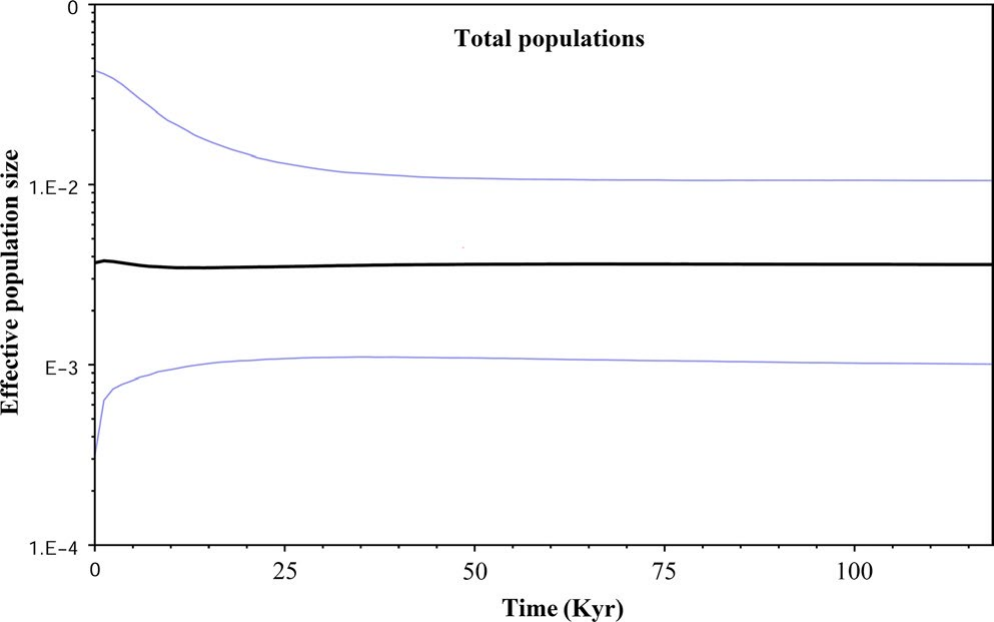
Bayesian skyline plots (BSPs) for *Neurergus derjugini*. BSPs derived using a mitochondrial ND4 sequences mutation rate of 0.64 per million years (Myr). The median estimates are shown as blue lines, and the 95% HPD limits are shown by the width areas between blue lines

The mismatch distribution for the *N. derjugini* appeared smooth and unimodal, consistent with a model of population expansion (Figure 5). The Harpending's Raggedness Index (*r* = .08, *p*(*r*) = .1) and sum of squared deviations (SSD) (*r* = .03, *p*(*r*) = .1) were not significant, indicating population expansion. Results of Tajima's D test was positive and not significant (0.60, *P*(*r*) = .10). However, negative value (−0.63, *P*_Fs_*__* =  0.04) for Fu's Fs, which is more sensitive to recent population expansions, suggested the recent demographic expansion. Bayesian skyline plots suggest that population size was increased very slightly 25 kyr (around LGM), (Figure 6). BSP_s_ showed a decline of population size about 2.5 kyr. Thus, the BSP_s_ suggest that populations went through a bottleneck in the cold period around 2.5 kyr.

### 3.5 Environmental data

**Figure 7:**
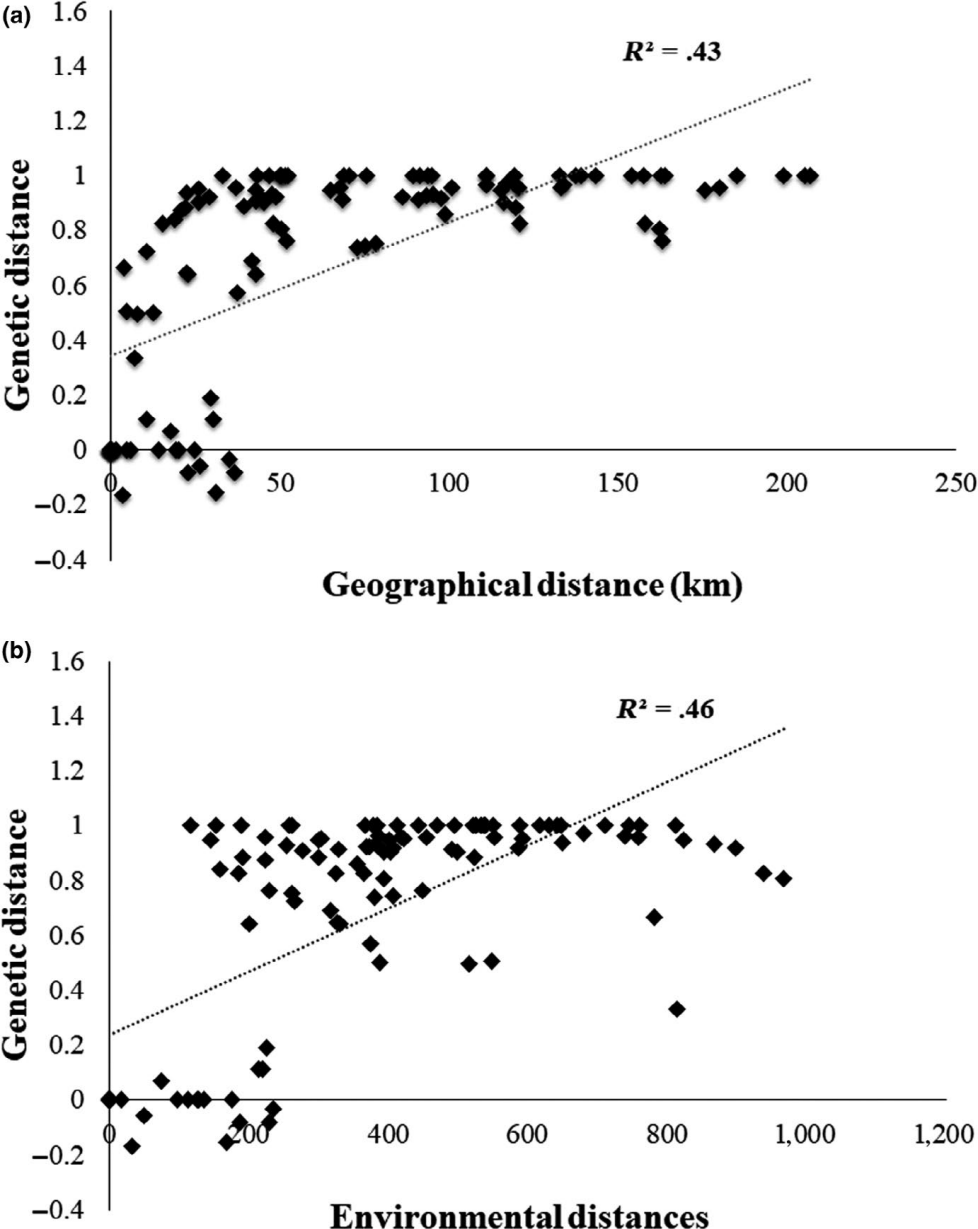
The plot of simple Mantel test showing the relationships between geographical (a) and environmental distance with genetic distance (b) among 15 populations of *Neurergus derjugini*

The analyzed populations revealed significant levels of isolation by distance (*r* = .66, *p* < .0001; Figure 7a) and correlation between genetic divergence and environmental distance (*r* = .68, *p* < .0001; Figure 7b). Mantel test detected a signal of isolation by environment had significant influence on genetic distance. Each of two variables in isolation had a significant effect on genetic differentiation. In the three‐way Mantel test, the correlation between genetic and geographical (*r* = .36, *p* = .0068) and environmental distances remained significant (*r* = .30, *p* = .0069).

**Figure 8:**
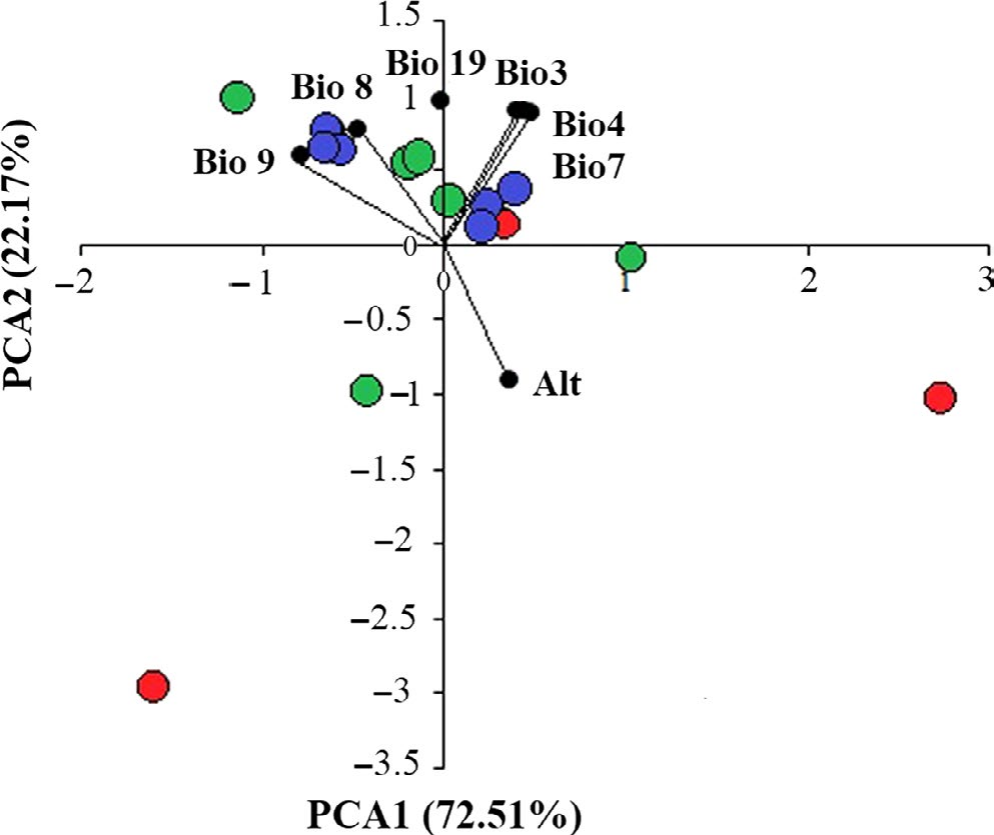
Plot of PCA based on environmental variables describing *Neurergus derjugini* localities. The populations in the northern (red circles), central (green circles), and southern (blue circles) regions were not separated along the PC1 and PC2 describing 15 *Neurergus derjugini* localities

The first two principal components (PC1 and PC2) explained 72.51% and 22.17% of the total variation and did not separate the northern, central, and southern localities along temperature and precipitation gradients (Table 6, Figure 8). The occurrence sites were not divided into separated environmental spaces in the Cartesian coordinates formed by the first two principal components (Figure 8).

**Table 6:** Pearson correlation coefficients between environmental variables and principal component axes describing 15 *Neurergus derjugini* localities

Environmental variables	PCA1	PCA2	PCA3
BIO3			
Isothermality	0.902	0.404	0.027
BIO4			
Temperature seasonality	0.879	0.473	0.048
BIO7			
Temperature annual range	0.895	0.442	0.03
BIO8			
Mean temperature of wettest quarter	0.781	−0.473	0.402
BIO9			
Mean temperature of driest quarter	0.595	−0.784	−0.086
BIO19			
Precipitation of coldest quarter	0.959	−0.015	−0.182
Alt			
Elevation	−0.897	0.363	0.202
Eigenvalue	5.076	1.552	0.246
% of variance	72.514	22.17	3.521

### 3.6 Divergence time estimate

**Figure 9:**
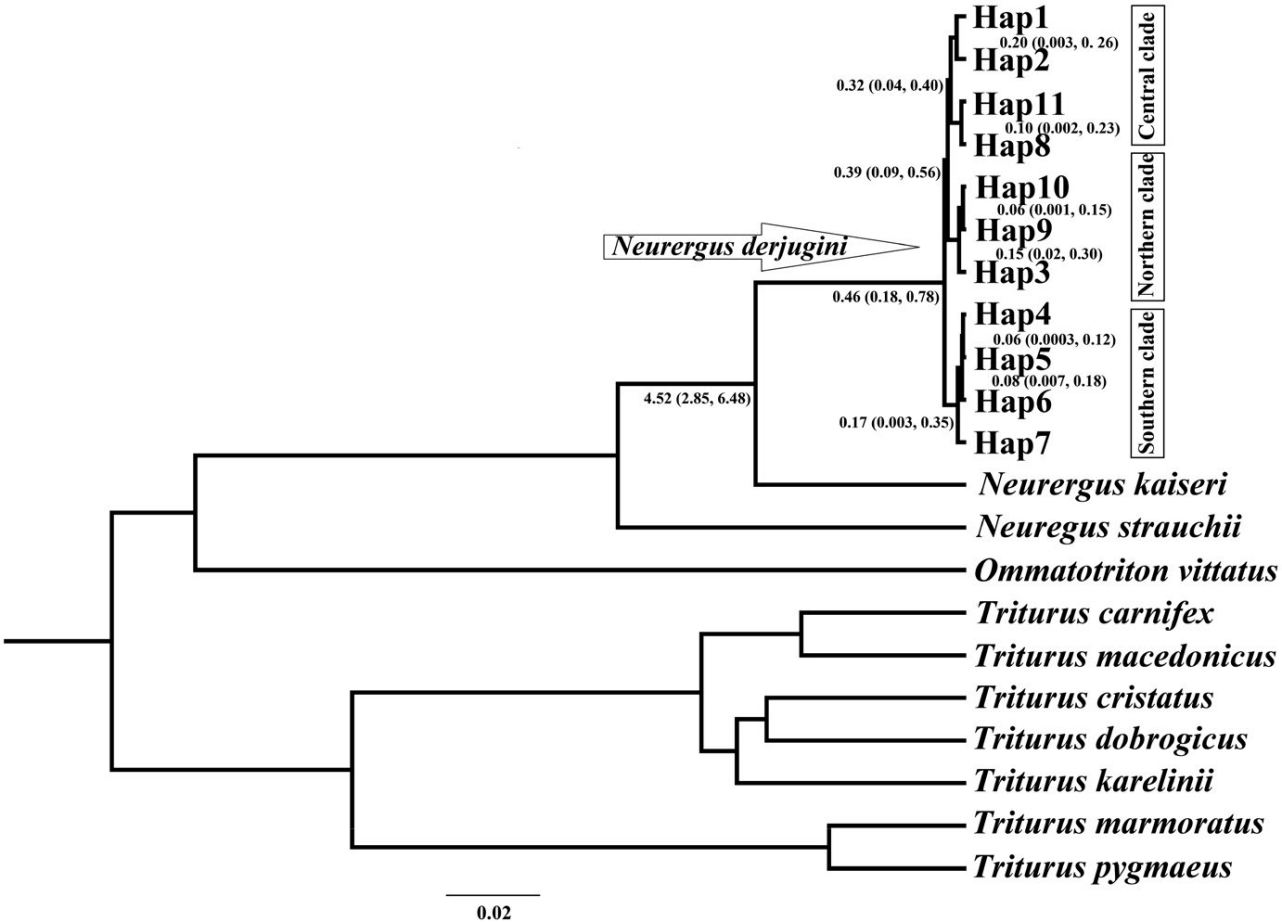
Chronogram of diversification implemented in BEAST based on ND4 gene for *N. derjugini*. The divergence times with 95% highest posterior density (95% HPD) are presented below the branches

Dating of major mtDNA cladogenetic events within *N. derjugini* haplogroups is presented in Figure 9. The Bayesian estimate of the age of mtDNA diversity in *N. derjugini* between the north‐central haplogroups and southern haplogroups was 0.46 Myr and divergence between northern and central haplogroups took place in 0.39 Myr (middle Pleistocene). Evolutionary ages of clades north and south range from 0.15 to 0.17 Myr, while the age of clade center is higher, corresponding to 0.32 Myr.

## 4 DISCUSSION

The Yellow‐spotted mountain newt exhibited a complex phylogeographic pattern with multiple divergent mtDNA clades across its relatively small range (6,366 km^2^). A total of 3 allopatric and 11 parapatric clades were identified for ND4, most of them with a very restricted distribution in northern, central, and southern parts of the distribution. Of 11 haplotypes identified by ND4 in present the study, 3, 4 and 4 haplotypes are reciprocally monophyletic for northern, central, and southern sections of the species range. Previous studies on monophyly in *N. derjugini* as evidence for refugia matching include six haplotypes identified in the control region (D‐loop) which form a well‐supported monophyletic clade within northern and southern populations (Afroosheh et al., 2019) and 11 haplotypes identified in ND2 with three monophyletic clade each 3, 3 and 5 haplotypes in north, center, and south (Salehi, Akmali, & Sharifi, 2019).

The present phylogeographic structure of *N. derjugini* does not match to the current subspecific division of this species as reported by Nesterov, (1917) and Hendrix et al. (2014): *Neuregus derjugini derjugini* and *N. d. microspilotus.* Frequently, number and size of yellow spots on the dorsal section of body have been used to distinguish different forms of the Yellow‐spotted mountain newts (Schneider & Schneider, 2011). However, use of this feature in taxonomy of the Yellow‐spotted newts in Middle East has caused uncertainty because more than one species of newt in Iran and neighboring Iraq and Turkey have yellow spots (Schneider & Schneider, 2011; Sharifi, Naderi, & Hashemi, 2013). Although a taxonomic revision is beyond the scope of this study, our results indicate that the current intraspecific taxonomy reflects local phenotypic varieties rather than distinct evolutionary units. According to a study conducted by Vaissi, Parto, and Sharifi (2018), on captive *N. derjugini* spot configuration (numbers, circularity, size, and asymmetry) change drastically during first three years before sexual maturation and remain fairly constant thereafter. In another study on configuration of yellow spots in a free living *N. derjugini* based on photo‐identification method a complete spatial randomness (CSR) test using nearest neighbors distances for the spots over dorsal part in adult newt showed a reasonable match with the Poisson distribution indicating that spots are distributed randomly over body of the newt (Sharifi et al., 2013). If such differences prevail, spot features are not adequate external characteristics to firmly determine taxonomic status of various populations of the species groups unless differences in the number, size, and also their stability are determined throughout the distribution range of the species. Therefore, any new arrangement within species of the genus *Neurergus* should be based on detailed intrapopulation examination of morphological features, ontogenetic pattern development, and genetic characteristics of different populations (Vaissi et al., 2018).

The finding of present genetic divergence among geographical clusters of populations of *N. derjugini* has not provided an indication for the existence of cryptic species because the degree of genetic divergence found between various haplogroups are within the range of the distances found in other accepted newt species. Although there appears to be a lack of evidence for sympatric deviations between mitochondrial lineages, it might be suggested that some form of barrier to gene exchange has existed between the distinct lineages. Steinfartz, Hwang, Tautz, Öz, & Veith, 2002, based on two sections of the 12S and 16S mitochondrial ribosomal genes (810 bp), 19 allozyme and three plasma protein loci, showed that intrageneric distances (HKY‐Г0.5 distances) for *N. strauchii strauchii* and *N. s. barani* was 1.4%. Also, Özdemir, Üzüm, Avci, & Olgun, 2009 used two mitochondrial genes (478 bp of 12S rRNA and 355 bp of 16S rRNA) from *N. crocatus*, *N. strauchii strauchii,* and *N. s. barani* and found two subspecies of *N. strauchii* differed by 4.5%–5.1% (*N. strauchii strauchii*) and 5.4%–5.5% (*N. s. barani*) of the pairwise uncorrected p‐distances of *N. crocatus*. The p‐distances between these two subspecies of *N. strauchii* ranged from 0.48% to 1.2%. Pairwise uncorrected p‐distances between clades in present study range from 0 to 0.002 in ND4 and from 0 to 0.004 in ND2 and 0 to 0.001 in D‐Loop. Average values of p‐distance for these genetic markers is not a level of divergence typically found between recognized species of the genus *Neurergus*; therefore, there may not be adequate evidences to support that *N. derjugini* constitutes a species complex.

The phylogeographic pattern observed in present and two more studies (Afroosheh et al., 2019; Salehi et al., 2019) on population genetic structure of *N. derjugini* with three mitochondrial markers have yielded two (D‐Loop) and three (ND2 and ND4) reciprocally monophyletic lineages. Investigation on mitochondrial DNA variation and reconstruction of the potential current and past distribution of *N. derjugini* together with landscape connectivity analysis showed higher gene flow between the breeding streams in the southern part of the range, while the northern populations are more isolated (Afroosheh et al., 2019). This study also showed that during the mid‐Holocene and LGM conditions, the range of the species may have been more extended and shifted to lower elevations. These findings show retraction of the *N. derjugini* range during the Quaternary and indicate that range dynamics of the species occupying lower latitudes where they would have a better chance to find glacial refugees (Afroosheh et al., 2019). A reasonable explanation for this phylogeographic pattern in an area with complex topography in mid‐Zagros range is one of the larger numbers of refugia occurring in the south to benefit from warmer climate close to the Mesopotamian Plain at lower elevation in the LGM. Phylogeographic analyses on many American and North European species have demonstrated the classic pattern of “southern genetic richness and northern purity” (Fritz et al., 2012, 2008, 2009). Contrarily to this configuration, the pattern of distribution of genetic diversity in *N. derjugini* is an exception.

To our knowledge *N. derjugini* is the first reported case of differentiation into multiple refugia within Zagros range in western Iran. The occurrence of allopatric lineages in northern, central, and southern species range (Figures 2, 3 and 9) which are reciprocally monophyletic provides evidence for a long‐term persistence of *N. derjugini* in these areas during Pleistocene glaciations, within separate refugia. Although, the phylogeographic pattern of *N. derjugini* fits the classical southern refugia model this study cannot show if *N. derjugini* used these areas as both glacial refugia and the source areas for northward postglacial colonization. Considering that the highest genetic diversity of these lineages is found in the southern portion of the species range it can be hypothesized that the refugia where these lineages differentiated were located in proximity of this area. In present study, variation in ND4 has revealed notable levels of genetic structuring and a low nucleotide diversity in *N. derjugini*. However, haplotype diversity for the total populations was relatively high. Low nucleotide diversity associated with a relatively high haplotype diversity in species provides evidence for a recent and rapid population expansion (Spear, Peterson, Matocq, & Storfer, 2005). However, it may also be due to the limitation caused by using a single‐genetic marker and limited sampling (Wang, Jiang, Xie, & Li, 2013).

There are several studies on salamanders and newts that follow multiple refugia scenario. For example, Church, Kraus, Mitchell, Church, and Taylor (2003) analyzed two mitochondrial markers of individuals of the eastern tiger salamander, *Ambystoma tigrinum tigrinum*. The results appear to be multiple Pleistocene refugia with little migration among the remaining populations. Sotiropoulos et al. (2007) performed phylogenetic analyses of *Mesotriton alpestris* populations from the entire range of species distribution, using fragments of two mtDNA genes. Extensive sequence divergence, implying greater isolation in multiple refugia, is found within eastern clades, while the western clades seem to have been involved in the colonization of central, western, and northeastern Europe from a hypothetical refugium in central Europe. Mattoccia et al. (2011) analyzed spatial variation of mtDNA sequences, aiming to investigate the genetic structure within each species, as well as attempting to trace the recent demographic and phylogeographical history of the genus *Salamandrina* in Italy. In this study, three main southern European refugia areas have been identified, corresponding to the Iberian, Italian, and Balkan Peninsulas.

The presence of several monophyletic groups indicate the existence of multiple refugia and has been used for explanation of genetic structure in many amphibian species (Giovannotti, Nisi‐Cerioni, & Caputo, 2010). The pattern of haplogroups in present study is, also, confirmed by AMOVA analysis, showing a pronounced geographical partition of *N. derjugini* genetic variation. One reason for a highly structured genetic diversity in populations occurring in close distances may be high fidelity to small home ranges in breeding streams reported for many species of amphibians including *N. derjugini*. Using photographic identification method Sharifi and Afroosheh (2014) showed that the average minimum distance covered by recaptured individuals in this species was only 49.19 ± 71.75 m. This value shows that the home range of *N. derjugini* in the breeding streams was estimated to be only 230 m^2^ (Sharifi & Afroosheh, 2014). Although these data show that *N. derjugini* has high fidelity to its aquatic environment, there is no evidence showing fidelity to either foraging grounds or to overwintering habitats.

A study on species distribution modeling of *N. derjugini* during current, the mid‐Holocene (6 ka BP) and the Last Glacial Maximum (LGM, 21 ka BP) shows that the potential distribution area of *N. derjugini* during the last LGM is characterized by low elevations and dry habitats (Afroosheh et al., 2019). This model anticipates that the species may have expanded north‐westwards during mid‐Holocene and it may have been moved to lower elevations during LGM (Afroosheh et al., 2019). This study, also, showed that according to Circuitscape analysis, northern populations have become more isolated while the southern populations have more connectivity. The model showed main potential refugia in the Zagros mountain system. These potential refugia are more concentrated in the central part of the species distribution range where central clade is present as the oldest clade with divergence time 0.32 Myr. This study, also, indicates that during the LGM and mid‐Holocene, the range of the species may have been more extended and shifted to lower elevations where they would have changed around to find glacial refugees. In present study, the monophyly of the *N. derjugini* populations has been occurred in main refugial areas. The presence of geographically structured clades by AMOVA analysis indicated the disjunct populations of *N*. *derjugini* may have been survived in three different glacial refugia in the Zagros mountains. Furthermore, the phylogenetic analyses based on another mitochondrial gene (ND2 and D‐loop) in *N. derjugini* (Figure 3) confirmed the presence of three reciprocally subclades and multiple glacial refugia.

## 5 CONCLUSIONS

Phylogeographic analysis based on mitochondrial markers in various populations of *N. derjugini* provides evidence for a history of isolation and divergence in allopatry resulting in the diversification of three monophyletic and geographically separate clades. These diversifications are largely concordant with the last glacial maxima. Positive and significant correlation between geographical, environmental, and genetic distances suggests a possible impact of geographical and environmental divergence in shaping the genetic variation of *N. derjugini* that have emerged in three different refugia. Principal component analysis did not separate climatic profiles of the distribution range of *N. derjugini* and demonstrated the homogeneity in different bioclimatic variables in the distribution range of *N. derjugini*. The populations occurring in northern, central, and southern sections of the species range have been separated from one another since 0.46 Myr between the north‐central haplogroups and southern haplogroups and 0.39 Myr between northern and central haplogroups took place in middle Pleistocene. Results obtain from current study, also, signifies the role of refugia in conserving populations as well as genetic diversity of *N. derjugini*.

## CONFLICT OF INTEREST

The authors declare that they have no competing interests.

## AUTHORS' CONTRIBUTIONS

SV and MSH conceived the main idea with input from MM and SV collected the data; and MM and SV analyzed the data with support from MSH. Sampling was done by SV and MSH. The manuscript was written by SV, MSH, and MM. All authors made substantial contributions to the interpretation of results and the editing of the manuscript.

## ETHICS APPROVAL AND CONSENT TO PARTICIPATE

This study complied with the appropriate institutional, national, and international guidelines.

## Data Availability

MtDNA haplotypes used in this study were deposited in the NCBI Nucleotide Database under accession numbers MN995069 to MN995079.
